# The more IGRT systems, the merrier?

**DOI:** 10.1002/acm2.12126

**Published:** 2017-06-26

**Authors:** Baozhou Sun, Jenghwa Chang, Yi Rong

**Affiliations:** ^1^ Department of Radiation Oncology Washington University St. Louis MO USA; ^2^ Department of Radiation Medicine Northwell Health and Hofstra Northwell School of Medicine at Hofstra University Lake Success NY USA; ^3^ Department of Radiation Oncology University of California Davis Comprehensive Cancer Center Sacramento CA USA

## 1 INTRODUCTION

The field of radiotherapy (RT) has benefited substantially from advancements in Image‐Guided Radiotherapy (IGRT) in the past 15 years. IGRT now constitutes the integration of a wide range of imaging technology with modern RT delivery systems that include 3D anatomical and functional‐based imaging for tumor volume identification, 3D target volume localization, and motion management information for precise patient setup and monitoring.^1^, ^2^ To streamline this complex process, system integration of planning and delivery with multimodality IGRT technologies is now a primary selling point for vendors. This integration becomes more complex with the increased number of image‐guided patient positioning and motion management options. Current IGRT technologies include not only various x‐ray based imaging systems but also other modalities, such as video/infrared (IR) cameras, ultrasound (US), and electromagnetic field systems. The capital purchase decision makers at hospitals welcome tools that allow for improved image guidance when it is consistent with their strategies for return on investment. But this may raise multiple issues that need to be addressed by medical physicists, including safe and practical implementation and commissioning, personnel qualification and training of staff, updates and servicing to ensure integration between systems, and of course reimbursement constraints. This brings us to our debate topic: Will more IGRT systems implemented in the clinic lead to better outcomes for RT treatments?

Arguing for the proposition is Dr. Baozhou Sun. Dr. Sun is an assistant professor and chief of quality assurance services of radiation oncology at Washington University in St. Louis. He earned his Ph.D. in applied science from the College of William and Mary in 2005. Dr. Sun finished his medical physics residency training at Washington University in St. Louis in 2012 and became a faculty member at the same institution. He is certified in Therapeutic Radiological Physics by the American Board of Radiology. His research interests include quality assurance, proton therapy, imaging‐guided radiation therapy, and medical informatics.

Arguing against the proposition is Dr. Jenghwa Chang. Dr. Jenghwa Chang received his Ph.D. in electrical engineering from Polytechnic University and is an ABR‐certified medical physicist. He is currently an Associate Professor at Radiation Medicine of Northwell Health supervising the training/education of medical/physics residents and overseeing the quality assurance program for physics. Previously Dr. Chang held positions with Weill Cornell Medical College, NYU Langone Medical Center, and Memorial Sloan‐Kettering Cancer Center. He is also a physicist surveyor for ACR Radiation Oncology Practice Accreditation (ROPA) program. His research interest includes optical diffusion tomography, Electronic Portal Imaging Device (EPID) dosimetry, MV/kV cone beam CT (CBCT), magnetic resonance imaging (MRI)‐guided treatment planning, panoramic CBCT, and setup uncertainty of single isocenter for multiple targets technique.

## 2 OPENING STATEMENT

### 2.A Dr. Baozhou Sun

The goal of RT is to deliver high dose to the tumor while sparing adjacent normal healthy tissues. The geometric accuracy of dose deposited to the desired target is critical to ensure high quality of treatments. IGRT has been introduced to reduce geometric uncertainties in RT. The diverse technologies of IGRT have been proved to be an effective quality control process that reduces the systematic and random uncertainties in the treatment process.^3^ In the era of precision and personalized medicine, more IGRT technologies should be developed and implemented to provide more benefits to patient care. The overall benefits of these IGRT technologies can be summarized in the following aspects:

1. Treatment margin reduction. During the planning process, in order to ensure adequate coverage of the clinical target volume, a margin has to be added to compensate the daily positioning uncertainties and internal organ motion. With the introduction of IGRT, the margins can be significantly reduced, leading to a substantial reduction in the normal tissue irradiation.
2. Hypofractionated RT targeting accuracy**.** Advances in IGRT has enabled hypofractionated RT and stereotactic RT (SBRT or SRS), which reduces the cost from conventionally fractionated RT and has clinically demonstrated superior benefits to conventional treatments in specific disease sites, such as lung, brain, liver, and prostate.
3. Anatomy change monitoring and adaptive RT. With IGRT, the daily or real‐time imaging data can be used to monitor changes in tumor size and shape over a course of treatment and make offline or online adaptations to the treatment plan. Both complex geometric errors and patient‐specific variation (e.g., tumor shrinkage) can be corrected through adaptive planning.

Overall, the use of IGRT in improving treatment margin reduction, hypofractionated RT accuracy, and adaptive RT will ultimately improve clinical outcomes.^4^, ^5^

It is without questions that IGRT is critical for ensuring treatment quality of RT. Currently, there exist a variety of commercial IGRT technologies readily available for clinical use. Different IGRT systems are used for different clinical scenarios depending on the treatment sites, expected magnitude of errors, and purpose of the application (positioning, target localization, or real‐time tumor tracking). The IGRT systems can be generally divided into radiation‐based and nonradiation‐based systems. Each system has its unique advantages and limitations when implemented in the clinic. The radiation‐based systems mainly use kV or MV imaging techniques and include:

1. 2D planar imaging using MV EPID and kV on‐board imager (OBI). Both EPID and kV OBI imaging systems are standard IGRT equipment for almost all linacs. These images are lack of soft tissue contrast, but provide bony landmarks as an aligning surrogate. The 2D kV with fiducials can also be used for tumor tracking for robotic radiosurgery system.
2. 3D volumetric imaging technology (CBCT, MV helical CT, and in‐room helical CT). CBCT provides better contrast resolution than MV helical CT (e.g., tomotherapy) and is the most commonly used system for daily localization on modern linacs. Image quality of MV CT is inferior compared to kV CT, but MV CT can reduce metal artifacts, which is useful for patients with dental filling or prosthesis. In‐room CT or CT‐on‐rails has been developed for IGRT. However, it has not been adopted by many due to its bulky size, high cost, and impracticality to implement in a regular linac room. Recently, a more compact design of mobile CTs has emerged and implemented for proton therapy.^6^ CT‐on‐rails or mobile CTs can also be used with brachytherapy high‐dose rate (HDR) after‐loaders for image guidance brachytherapy.^7^

Nevertheless, the 3D volumetric imaging does not provide “snap shot” information and cannot be used for intra‐fraction monitoring or correction. With the advantages of no extra radiation to patients, the nonradiation‐based systems have also been widely implemented in RT, which include:

1. Camera‐based systems (e.g., surface monitor systems such as VisionRT or C‐Rad). These systems can be used for patient positioning and intra‐fraction monitoring. Yet they are mostly limited to situations where external surface acts as a reliable surrogate for internal position or motion.
2. Electromagnetic tracking (e.g., Calypso). This system uses electromagnetic transponders embedded within the tumor, and motion of these transponders may be tracked in real time using a detector array system. However, it is only limited to prostate.
3. US‐based system (e.g., BAT, Clarity). US has several advantages including relatively low cost, avoidance of invasive seed placement procedures, and the potential of reduced patient setup times. Sites of common application include prostate and breast.

During the last decade, the development of image guidance in the context of radiation therapy has been substantially accelerated. The recent development in MR‐guided radiation therapy has significantly advanced the field of IGRT. MR‐guided treatment machines have better contrast for soft tissue and provide real‐time assessment of internal soft‐tissue anatomy and motion and allow for intra‐fractional corrections.

There is no single system that can be applied for all clinical scenarios as a treatment machine is often used to treat multiple sites. Different treatment sites and modalities require different levels of accuracy. For example, a simple 2D or 3D treatments, MV portal imaging is sufficient for localization, giving the required level of setup accuracy is on the order of cm. While for targets adjacent to critical structures, daily kV or CBCT might be required to reduce setup uncertainties to the order of mm. For hypofractionated treatment (SBRT or SRS), real‐time tracking is considered ideal to monitor the intra‐fractional motion. Therefore, more IGRT technologies implemented in the clinic should bring more benefits for high‐quality treatments. For a large‐size RT clinic, there are usually dedicated machines with multiple IGRT systems for SBRT/SRS treatments. And integration of multiple IGRT systems into one single room is available to provide more flexibility and better performance. There are several studies on integration of 3D US to CBCT for prostate treatments.^8^, ^9^ For example, some newly released linac products are equipped with MV, kV 2D/2D, CBCT, respiratory gating, and optical surface monitoring systems. There is no doubt that more IGRT technologies implemented will lead to better geometric precision for treatments, which will provide more benefits to patients and improve the quality of patient care.

### 2.B Dr. Jenghwa Chang

Ever since the in‐room kV imaging system became commercially available at the beginning of this millennium, there has been an explosion of new IGRT systems introduced into the treatment room. These systems adopt complex combinations of imaging sources, detectors, and processing algorithms. Recently, one of vendors’ favorite marketing points for radiotherapy treatment machines is the “IGRT integration” with all possible imaging modalities on board, such as kV/MV 2D projection imaging, kV/MV 3D volumetric imaging, surface‐based imaging, electromagnetic imaging, ultrasound. Moreover, MRI‐based IGRT systems combined with the Cobalt or linac treatment unit are on the horizon and promised to elevate the IGRT complexity to a whole new level. This IGRT technology push is based on the seemingly indisputable claim “more IGRT systems implemented in clinic are better for radiotherapy outcomes.” That is, more IGRT systems offer more treatment options so that the best technology or combination of technologies can be used to guide the treatment. This would allow us to see more details of patient anatomy, physiology, and pathology, which can lead to better tumor targeting and less normal tissue complications.

However, this improvement is usually achieved at the cost of increased complexity of the IGRT program. Specifically, therapists, radiation oncologists, and medical physicists might need to spend longer time to perform imaging scans, identify the treatment target, and correct patient's positioning. In addition, the longer we spend on IGRT processing and analysis, the more likely the patient's position has already changed since first imaged and might require a repeat of imaging, which further prolongs the IGRT procedure. The additional machine time and personnel time spent on these multilevel imaging studies need to be either paid by insurance companies or absorbed by hospitals. Finally, the people present at an IGRT procedure must be fully competent in the technology (or technologies) chosen for that procedure. In theory, these additional personnel, financial, and educational/training resources must be available when more IGRT systems are incorporated. However, as Yogi Berra pointed out, “In theory there is no difference between theory and practice. In practice there is.” What is the IGRT in practice?

First, insurance companies are limiting medical payments. The United States has the highest healthcare expenditure per capita in the world,^10^ and the trend of increase is not sustainable.^11^ In order to control the medical spending, insurance companies are cutting back the reimbursement rate, particularly for procedures employing advanced medical technologies. In the radiotherapy billing code system, bundled payments have been introduced in the past few years, so the payment for an IGRT treatment is fixed regardless of the complexity of the procedure. Therefore, the extra cost for IGRT, if there is any, needs to be absorbed by the hospitals.

Second, the machine and personnel times spent performing IGRT are often restricted. Currently, most clinics schedule a 15‐ or 30‐minute slot, respectively, for the regular fractionated or SRS/SBRT cases. These time slots are not always enough to perform all desired imaging studies for guiding the treatment, particularly for the patients who need longer time for image co‐registration of complex anatomy, or require frequent imaging to correct for significant intra‐fraction motion. Hospitals used to absorb the extra cost for procedures requiring longer treatment time but without getting additional reimbursement. However, in order to survive today's competitive healthcare environment, a radiotherapy clinic needs to maintain a sustainable patient throughput and schedule patients within the allowed time slot. As a result, most hospitals are reluctant to carry this financial burden.

Furthermore, personnel qualification for IGRT procedures is questionable. As identified by professional societies^12^ and accreditation bodies,^13^ one key factor to the success of an IGRT program is making sure the personnel has sufficient education/training with the IGRT systems and procedures. Ideally, education/training policy should be strictly enforced so that the IGRT is always operated and supervised by qualified therapists, medical physicists, and radiation oncologists. In reality, with more (including IGRT) technologies introduced into the field,^14^ it has become increasingly more difficult to maintain adequate education/training for the staff. Moreover, it is more challenging to interpret the results from a combination of various IGRT modalities (e.g., optical surface imaging combined with CBCT, or electromagnetic imaging combined CBCT) than from individual modality. However, most IGRT education/training focuses on the learning of individual system while the processing of mutual information between modalities is usually under‐addressed. Without extra training and knowledge, the staff might not feel comfortable making a clinical decision, particularly when the results from multiple imaging studies do not agree.

Finally, more IGRT systems also challenge the logistics of radiotherapy delivery. A radiotherapy room clogged with multiple IGRT systems is not only difficult for therapists to operate but also more likely to have treatment‐related incidents, e.g., collision, particularly for vaults built before the IGRT era. Moreover, therapists can be easily distracted by all the imaging equipment in the room and extra monitors on the treatment counsel. More IGRT systems also lead to longer time for morning QA, more scheduling conflicts for machine QA and maintenance, and extra competition for storage spaces. In addition, frequent personnel turnover and scheduling conflict might force the institution to staff a machine with inexperienced people. This situation is made worse by the increased use of per‐diem therapists and medical physicists (for cost‐cutting purpose) who usually have limited knowledge and experience on the IGRT program the institution has implemented. With all the risks added together, the safety of the radiation delivery might be compromised and the benefit of more IGRT technology is offset by possible increased errors.

In conclusion, complexity is the ultimate enemy. The statement “more IGRT systems implemented in clinic are better for radiotherapy outcomes” is only true in theory but could not materialize in practice because most clinics do not have enough machine time and man power to deal with the added complexity. Even worse, implementing more complex IGRT procedures without sufficient resources might actually lead to more mistakes and eventually degrade the quality of radiotherapy program.

## 3 REBUTTAL

### 3.A Dr. Baozhou Sun

I do not agree with the statement “The additional machine time and personnel time spent on these multilevel imaging studies need to be either paid by insurance companies or absorbed by hospitals.” The availability of multiple imaging technologies does not always increase the additional personnel and machine time, but will increase the flexibility of IGRT for different sites. Users do not need to take multiple imaging scans for each patient, but instead, select the best IGRT technique based on treatment sites or requirement of accuracy. Furthermore, as I described in the opening statement, with advanced IGRT technologies for tumor localization and real‐time tracking, SBRT has been widely accepted and implemented. With multiple advanced IGRT techniques implemented, more and more patients may be eligible for SBRT treatments. SBRT treatments, facilitated by IGRT, can reduce the treatment fractions from 35 to 3–5 fractions or even to a single fraction, which significantly reduces the machine time, personnel time, and overall cost.

With regard to the personnel qualification for IGRT procedures, I agree that personnel should have adequate training before a new IGRT technology is implemented. However, that does not draw the conclusion that we do not need to implement more IGRT technologies. IMRT or VMAT techniques are much more complex than 3D Conformal RT. It is incorrect that we discourage the implementation of IMRT or VMAT in the clinic due to its complexity or ignore the facts that IMRT or VMAT can provide better clinical outcomes than conventional 2D or 3D treatments. In the same way, multiple IGRT technologies result in more complicated RT treatments with better quality. Lack of training is the ultimate enemy, complexity is not. Complexity of multiple IGRT technologies can be managed by appropriate training, in order to ensure that the benefits of diverse IGRT are maximized to provide the best quality of patient care.

The “opposed” statement suggests that more IGRT technologies challenge the logistics of RT delivery due to clogged space, more time for morning QA, and therapist's distraction because of more IGRT equipment. However, we should note that advanced treatment machines provide integrated IGRT technologies. The radiographic, fluoroscopic, kV CBCT, and MV CBCT are seamless integrated in modern linacs. Those optical camera systems, i.e., VisionRT, are ceiling mounted and take little space. The workflow are efficiently designed and integrated in many advanced RT systems. Comprehensive and automated QA tools are available for imaging QA and can prevent errors due to multiple imaging technologies.

### 3.B Dr. Jenghwa Chang

In theory, I would agree with Dr. Sun's opening statement, particularly, “Overall, the use of IGRT … will ultimately improve clinical outcomes.” But this statement might not hold in clinical practice. I have gone over in my opening statement the reality of the healthcare industry and the obstacles a clinic might face when trying to implement multiple IGRT systems beyond necessity or its capacity. In this rebuttal, I would like to focus on a few points brought up by Dr. Sun.

First, in the era of value‐based medicine, in addition to the goal of achieving “better clinical outcomes,” another aspect should also be considered: are these aggressive and expensive treatments with high demands in IGRT technologies really necessary? Are there less‐expensive alternatives that can attain equivalent or better clinical outcomes? These questions are generally not answered and a couple of clinical trials are looking into them. For example, RTOG 0631 compares the treatment outcomes of 16 Gy or 18 Gy single‐fraction SRS of spine metastasis under high‐precision (better than 2 mm) image guidance with 8 Gy single‐fraction 2D or 3D external beam treatment with generous (1–2 cm) PTV margin.^15^

Moreover, successful implementation of multiple IGRT systems in a few large academic centers may not be directly translated to stand‐alone clinics or community hospitals that constitute the majority of oncology practices in the United States.^16^ This is mostly due to the differences in available resources and billing code systems. Large academic centers usually provide more research time and funding support to physician and physics faculties for investigating and experimenting new IGRT technologies. Stand‐alone clinics or community hospitals, however, have very limited financial, personnel, and training resources for managing even the basic clinical IGRT systems.

Finally, technology is the most significant contributing factor to the growth in healthcare spending, and it is essential to attain good value for money spent in technology development.^17^ The “substantially accelerated” pace in developing more complex IGRT technologies needs to take a breath. Instead, more efforts should be spent on developing IGRT systems that are simpler, more robust, and less expensive but can still provide good values. Until then, the statement “more IGRT systems implemented in clinic are better for radiotherapy outcomes” is only true in theory but not in practice.

## 4 CONFLICT OF INTEREST

The authors declare no conflict of interest.
